# Interstitial lung disease in systemic sclerosis—a retrospective cross-sectional study in Taiwan

**DOI:** 10.1007/s10067-025-07913-y

**Published:** 2026-01-07

**Authors:** Chih-Wei Liu, Yen-An Chang, Chia-Li Yu, Hung-Cheng Tsai, Wei-Sheng Chen, I.-Cheng Ho, Yi-Syuan Sun, Hsien-Tzung Liao, Chang-Youh Tsai

**Affiliations:** 1 Division of Allergy, Immunology and Rheumatology, Taipei Municipal Gan-Dau Hospital (Managed By Taipei Veterans General Hospital), Taipei, Taiwan; 2 Division of Allergy, Immunology and Rheumatology, Department of Medicine, Taipei Veterans General Hospital, Taipei, Taiwan; 3 Division of Allergy, Immunology and Rheumatology, Department of Medicine, National Taiwan University Hospital, Taipei, Taiwan; 4 Faculty of Medicine, National Yang Ming Chiao Tung University, Taipei, Taiwan; 5 Division of Rheumatology, Inflammation, and Immunity, Department of Medicine, Brigham and Women’s Hospital, Harvard Medical School, Boston, U.S.A.; 6 Division of Allergy, Immunology and Rheumatology, Department of Internal Medicine, School of Medicine, College of Medicine, Taipei Medical University, Taipei, Taiwan; 7 Division of Immunology and Rheumatology, & Faculty of Medicine, Fu Jen Catholic University Hospital, Fu Jen Catholic University, New Taipei City, Taiwan

**Keywords:** Interstitial lung diseases (ILD), Mortality, Pulmonary function test, Pulmonary hypertension, Systemic sclerosis (SSc)

## Abstract

**Introduction/Objectives:**

Interstitial lung disease (ILD) is a crucial manifestation of systemic sclerosis (SSc), which has not been studied much in Asians.

**Methods:**

The electronic medical records of 110 SSc patients between April 2000 and December 2020 were comprehensively examined, including clinical manifestations, laboratory tests, pulmonary functions, and high-resolution computerized tomography (HRCT) of the chest.

**Results:**

Among 26 men and 84 women with a median age of 61 years in SSc cohort, 52 (47.3%) presented with ILD. The actual prevalence of ILD in SSc cohort was estimated between 10.5% and 47.3%. The most common form was usual interstitial pneumonitis (UIP). The total lung capacity (TLC)/diffusion capacity of carbon monoxide (DLCO), renal function, and CRP were poorer in the SSc–ILD who smoked less than the counterpart without ILD. A weighted score incorporated from essential parameters (risk of ILD = TLC − 0.1 × ESR − 1.5 × CRP) has modest power (ROC AUC 0.7242) to predict ILD. The main causes of death in the SSc–ILD were infections (72.7%), pulmonary hypertension (PH, OR = 18.81, 95% CI = 2.11–167.70), and renal failure (OR = 33.6, 95% CI = 2.00–546.10).

**Conclusion:**

The SSc–ILD have lower TLC/DLCO, higher CRP, and poorer renal function than the SSc without ILD. Early-onset dyspnea, PH, and renal failure may be independent risk factors for mortality in SSc–ILD.

**Table:** 

**Key Points** • *110 SSc (M: F=26:84) were retrospectively analyzed for ILD, with 10.5–47.3% presenting with ILD (UIP the most).* • *Risk of ILD in SSc = TLC – 0.1 × ESR − 1.5 × CRP can apply in this cohort.* • *Death originated from infections, PH, and renal failure in addition to early-onset dyspnea in this cohort.*

**Supplementary Information:**

The online version contains supplementary material available at 10.1007/s10067-025-07913-y.

## Introduction

Interstitial lung disease (ILD) is a poor prognostic complication of systemic sclerosis (SSc), which is life-threatening [1]. Diagnosis of SSc–ILD is challenging, requiring multidisciplinary evaluations by rheumatologists, pulmonologists, and radiologists. It is uncommon but widely distributed geographically and presents heterogeneously [2]. However, studies in the UK found ILD occurred in 60% of SSc [3]. Another study in Canada showed up to 32.6% pulmonary involvement in SSc [4]. Moreover, a survey in EUSTAR showed an 85.5% prevalence of ILD in clinically recognized SSc [5]. It is a leading cause of death in patients with rheumatic diseases [6, 7]. In the past three decades, renal crisis decreased from 42 to 6%, but death from lung diseases increased from 6 to 33% in SSc [8]. In a recent large cohort study, ILD was the most common organ involvement in SSc (74%) and was also the leading cause of fatal infections (50%) [9]. Although the incidence of SSc–ILD in Asians is at least unlikely lower than in Caucasians, thorough and comprehensive clinical and epidemiologic studies are surprisingly lacking, albeit its tailored management appropriate for Orientals. How to recognize ILD in SSc earlier in Asians and to provide effective and appropriate therapies for them is paramount important.

The present investigation aimed to identify clinical predictors for ILD and its mortality in Taiwanese patients with SSc. Such predictors might enable early diagnosis and intervention to help reduce death in Oriental SSc patients.

## Methods

### Patients

We conducted a retrospective cross-sectional study from the database in Taipei Veterans General Hospital. A total of 110 consecutive adult SSc patients (≥ 20-year-old), with at least a hospitalization record in the Immunology/Rheumatology ward for diagnostic procedures or treatments, were retrieved from the electronic medical records (EMR) between April 2000 and December 2020. All patients carried an ICD-9-CM or ICD-10-CM code for systemic sclerosis, which was accredited by a rheumatologist (710.1 or M34). A diagnosis of SSc is made according to ACR criteria or the LeRoy and Medsger criteria [10, 11]. The retrieved patients who were diagnosed between 2014 and 2020 fulfilled the 2013 ACR/EULAR classification criteria for scleroderma [12]. Patients with morphea or linear scleroderma were excluded, but those overlapping with other connective tissue diseases or mixed connective tissue disease (MCTD) were included once they were fulfilling the criteria for scleroderma in the duration of case retrieving. From the EMR, we retrieved different systemic presentations or comorbidities, including peripheral vascular disease (Raynaud’s phenomenon [RP] and/or digital ulcers), pulmonary involvement (ILD and pulmonary hypertension [PH]), cardiovascular diseases (CVDs), or gastrointestinal diseases (GIDs). ILD is defined by manifestations in plain X-ray and computerized tomography (CT) of the chest verified by radiologists. PH is defined as an estimated right ventricular systolic pressure (RVSP) of more than 36 mmHg [13], as determined by transthoracic echocardiography. Other CVDs, including hypertension (HTN), acute coronary syndrome, and/or congestive heart failure (CHF), were also collected from the EMR. GIDs included an abnormal esophageal transit time, gastro-esophageal reflux disease (GERD), esophagitis, gastric ulcers, and duodenal ulcers as demonstrated by upper gastrointestinal endoscopy, or colonic diseases as demonstrated by colonoscopy. Disease duration is defined as the time from diagnosis of SSc to ILD as shown in chest CT. The cause of death was collected from EMR according to the clinical diagnostic records if the patient had died in TVGH.

### Laboratory parameters

Laboratory data included hemoglobin (Hb), erythrocyte sedimentation rate (ESR), serum creatinine, albumin, C-reactive protein (CRP), complements C3 & C4, antinuclear antibodies (ANA), antibodies against anti-U1-ribonuclear protein (U1-RNP), topoisomerase I (Scl70), dsDNA, and SSA/SSB. Among these autoantibodies, U1-RNP, dsDNA, and SSA/SSB antibodies are for differentiation from MCTD, systemic lupus erythematosus (SLE), and primary Sjögren’s syndrome. The normal ranges of all laboratory parameters are provided by the individual equipment or test kit manufacturers.

### Pulmonary function tests

Functional lung assessments including forced vital capacity (FVC), total lung capacity (TLC), forced expiratory volume at 1 s (FEV1), and diffusion of carbon monoxide (DLCO) were performed according to the American Thoracic Society/European Respiratory Society guidelines, using standard equipment. FVC was measured using a water-sealed spirometer, and DLCO was obtained by a single-breath method corrected for hemoglobin.

### Statistical analysis

All data were analyzed using Statistical Package for Social Science (SPSS) Version 22.0 (Chicago, USA). Data were expressed as median and range. Comparison of continuous variables between two groups was assessed by Student’s *t*-test or Mann–Whitney *U* test, as appropriate. Comparison of proportions was assessed by chi-square test or Fisher’s exact test, depending on distributions of the variables. Two-sided *p*-values < 0.05 were considered statistically significant. Univariate and multivariate analyses were performed, with odds ratio (OR) and the corresponding 95% confidence intervals (CI) being calculated to determine independent risk factors associated with mortality.

## Results

### Demographic characteristics and autoantibody profiles in SSc patients

**Fig. 1:**
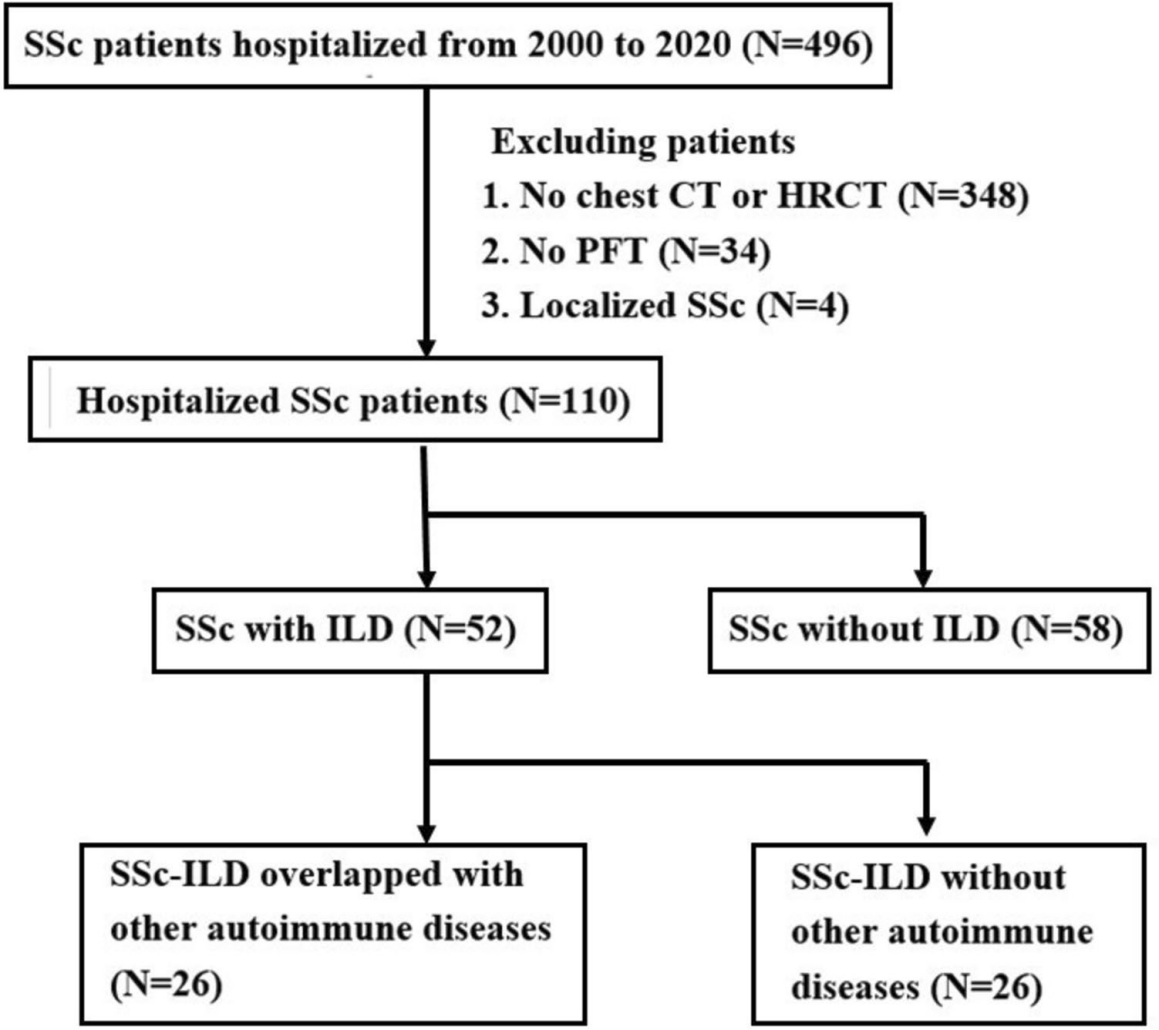
Study flowchart. Four hundred ninety-six SSc patients who were hospitalized during a period from April 2000 to December 2020 were included. Patients who did not undergo chest CT, HRCT, or PFT and who were diagnosed only with localized SSc were excluded. Finally, data from 52 SSc patients with ILD and 58 SSc patients without ILD were analyzed. SSc, systemic sclerosis; ILD, interstitial lung disease; CT, computed tomography; HRCT, high-resolution CT; PFT, pulmonary function test

**Fig. 2:**
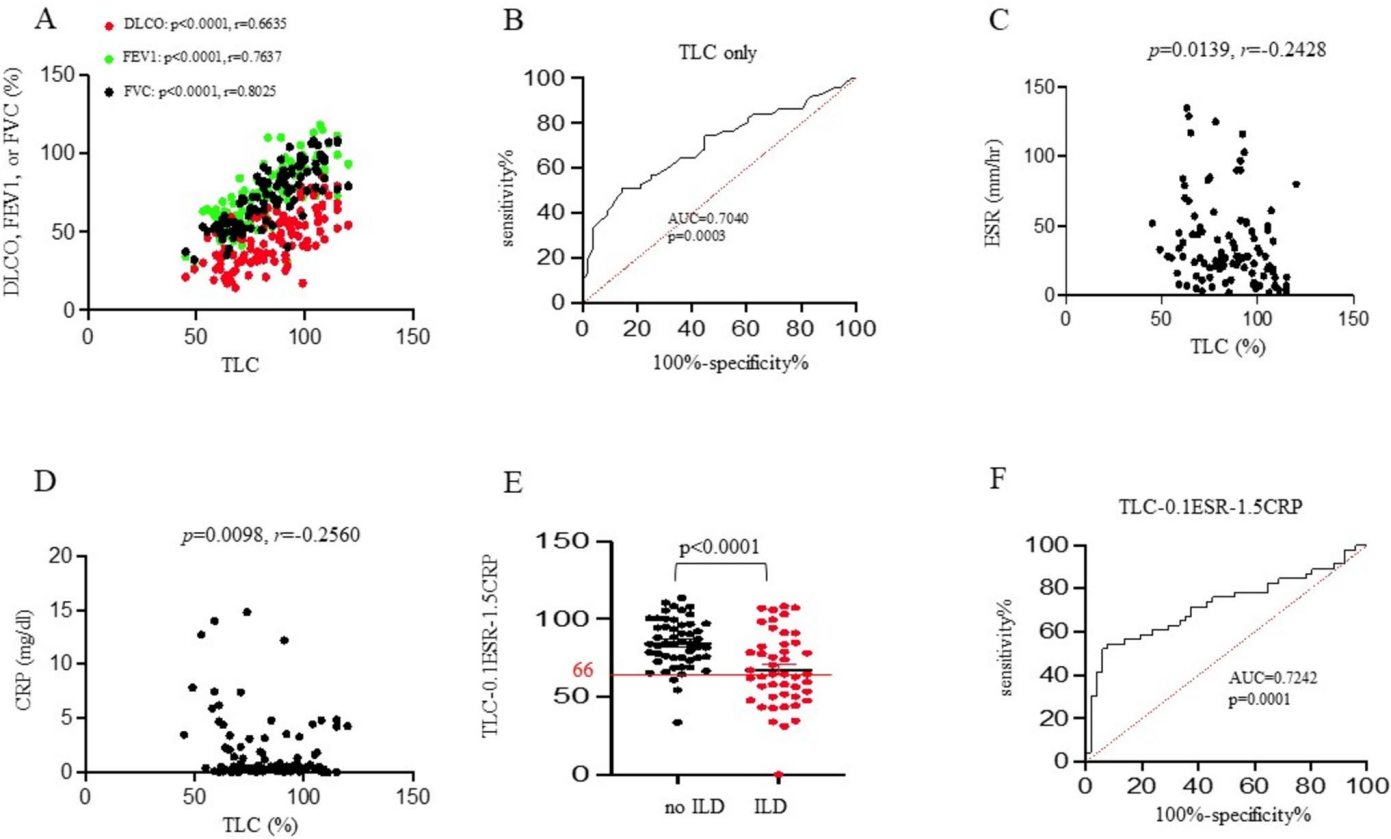
Clinical parameters and pulmonary function tests in study subjects. The correlation between TLC and other indicated PFT parameters of all study subjects is shown in **A**. The ROC curve comparing the raw TLC values between ILD and non-ILD is shown in **B**. The correlation between TLC and ESR and CRP of all study subjects is shown in **C** and **D**, respectively. The weighted scores (TLC – 0.1 × ESR – 1.5 × CRP) of ILD and non-ILD are shown in **E** and analyzed with ROC in **F**

Of a total of 496 patients with SSc and allied diseases enrolled from April 2000 to December 2020 (Fig. 1), 348 did not receive conventional chest CT or HRCT and thus were excluded from the study. Thirty-four patients who did not undergo PFT and/or DLCO and 4 who were recognized only with localized scleroderma were also excluded from the study, leaving 110 participants for the final analysis. So, a total of 386 patients are assumed to be free of pulmonary involvement. The demographic and clinical characteristics of the 110 patients are summarized in Table 1, indicating a female-to-male ratio of 3.2 (F:M = 84:26) and a median age of 61 years. Among them, 52 (47.3%) presented with ILD. Yet, taking into account the 386 patients not undergoing diagnostic procedures for pulmonary diseases, the prevalence of ILD in SSc patients may be lower (52/496 = 10.5%). However, because some of those 386 patients might have been lost to follow-up due to unknown reasons, not actually being free of lung involvement, the actual prevalence of ILD in our SSc cohort should be between 10.5% and 47.3%. The median age of onset was 45 years, and the overall mortality was 30.9% (34 patients). There were 48 (43.6%) overlapping with other autoimmune diseases, 85 (77.3%) presenting with Raynaud’s phenomenon, 79 (71.8%) using corticosteroids chronically, 98 (89.4%) having abnormal esophageal transit time, 94 (85.5%) having gastroesophageal reflux disease (GERD), 90 (81.8%) exhibiting serum antinuclear antibodies (ANA, > 1:80), but only 18 (16.4%) reporting a smoking history. The median age was 60.5 years when the diagnosis of pulmonary diseases was made according to the EMR, and the median disease duration from the onset of ILD was 4.5 years in the 52 SSc–ILD patients. Compared to those without ILD, they also had lower mortality (21.2% vs. 39.7%, *p* = 0.036), less smoking history (7.7% vs. 24.1%, *p* = 0.028), more decreased TLC of predicted (74% vs. 91%, *p* < 0.001) and FVC of predicted (65% vs. 81.5%, *p* = 0.001), lower serum creatinine level (0.73 vs. 0.84 mg/dl, *p* = 0.045), lower DLCO (40%, *p* = 0.005) and FEV1 (67.5% vs. 81.5%, *p* = 0.003), and a likelihood to have higher serum CRP (0.62 vs. 0.4 mg/dl, *p* = 0.059). In the medications for SSc, the frequency of steroid use did not differ between both groups. Expectedly, there is a strong positive correlation among the parameters of PFT (Fig. 2A). Among them, TLC carries the highest predictive value for ILD and alone has ROC AUC of 0.704 (Fig. 2B). The addition of other PFT parameters to TLC does not further increase the AUC value. In addition, there is a reverse correlation between TLC and the inflammatory markers ESR and CRP (Fig. 2C and D). A weighted score based on the equation.

**Table 1:** Demographic and laboratory characteristics of 110 patients with SSc

Clinical features	SSc with ILD (*n* = 52, 47.3%)		SSc without ILD (*n* = 58, 52.7%)		*p*-value
	*n* (%)	Median (range)	*n* (%)	Median (range)	
Age (years)		60.5 (31–89)		62 (28–86)	0.996
Female (%)	42 (80.8)		42 (72.4)		0.3
ILD after SSc diagnosed (year)		4.5 (0–32)			
SSc onset age (year)		43.5 (12–77)		48.5 (10–85)	0.168
Mortality (%)	11 (21.2)		23 (39.7)		0.036*
Smoking (%)	4 (7.7)		14 (24.1)		0.028*
Malignancy (%)	12 (23.1)		16 (27.6)		0.610
Overlapping autoimmune dz (%)	26 (50)		22 (37.9)		0.20
Digital ulcers (%)	21 (40.4)		19 (32.8)		0.40
PH (%)	20 (45.5)		18 (36.7)		0.393
Raynaud’s phenomenon (%)	44 (84.6)		41 (70.7)		0.18
Dyspnea (%)	34 (65.4)		31 (53.4)		0.2
ESR (mm/h)		28 (3–135)		23 (2–125)	0.153
CRP (mg/dL)		0.62 (0.01–14.8)		0.4 (0.01–14)	0.059
Creatinine (mg/dL)		0.73 (0.48–6.22)		0.84 (0.4–10.4)	0.045*
TLC of predicted (%)		74 (45–115)		91 (59–120)	< 0.001*
FVC of predicted (%)		65 (32–108)		81.5 (40–108)	0.001*
FEV1 of predicted (%)		67.5 (27–110)		81.5 (30–118)	0.003*
DLCO of predicted (%)		40 (17–79)		49.5 (14–85)	0.005*
Steroid (%)	34 (65.4)		45 (77.6)		0.16
Sildenafil citrate (%)	12 (23.1)		8 (14.5)		0.258
ANA (%)	43 (82.7)		47 (81)		0.343
Anti-Scl70 Ab (%)	22 (42.3)		18 (31)		0.122
Anti-RNP Ab (%)	9 (17.3)		11 (19)		0.768

*SSc* systemic sclerosis, *ILD* interstitial lung disease, *PH* pulmonary hypertension, *ESR* erythrocyte sedimentation rate, *CRP* C-reactive protein, *TLC* total lung capacity, *FVC* forced vital capacity, *FEV1* forced expiratory volume in one second, *DLCO* diffusing capacity of lung for carbon monoxide, *ANA* antinuclear antibody, *dz* diseases. *p-value < 0.05

$$Risk\;of\;ILD=TLC-0.1\times ESR-1.5\times CRP.$$

enables better differentiation of ILD from non-ILD (*p* < 0.0001) (Fig. 2E) and numerically increases the predictive power to AUC of 0.7242 (Fig. 2F). A score of 66 or lower has a sensitivity of 54% and a specificity of 90% in diagnosing ILD (Fig. 2E).

#### Causes of death in SSc patients with or without ILD

Thirty-four (30.9%) patients died in 110 SSc patients who underwent pulmonary evaluations. Among them, those without ILD accounted for more (39.7% vs. 21.2%). The overall main causes of death were infections (44.1%), cancers (29.4%), CVDs (20.6%), GI bleeding (5.9%), and large cerebral infarct (2.9%). In more detail, death in SSc patients who probably had lung diseases most likely originated from infection (72.7%), not necessarily confined to the lung. On the other hand, SSc patients without ILD succumbed more to malignancies (34.8%) and infections (30.4%) as compared to those with ILD. More data are detailed in the Supplementary Table S1.

#### Clinical features of the fatal SSc–ILD patients

Relevant clinical manifestations of the 11 mortal cases (21.2%) in SSc–ILD are presented in Table 2. The pertinent factors to the death included PH, CVD, early onset of dyspnea, decreased TLC and DLCO, increased levels of CRP, ESR, creatinine, and hypoalbuminemia. Dyspnea was observed in all 11 fatal patients (100%), while only 56.1% of the surviving SSc–ILD patients presented with this symptom. Similarly, PH was observed in 81.8% of SSc–ILD patients who died. As calculated by univariate logistic regression, PH (odds ratio (OR) = 18.81, *p* = 0.009), high ESR (OR = 1.22, *p* = 0.029), high CRP (OR = 1.23, *p* = 0.026), hypoalbuminemia (OR = 0.23, *p* = 0.01), increased serum creatinine (OR = 33.60, *p* = 0.014), diminished TLC (OR = 0.93, *p* = 0.021), and DLCO (OR = 0.86, *p* = 0.003) were associated (either increasing or decreasing) with mortality in SSc–ILD patients (Table 3). However, multivariate logistic regression analysis failed to reveal any significant differences (data not shown). Similarly, autoantibody profiles or steroid usage were not significantly related to the mortality in SSc–ILD patients.

**Table 2:** Comparison of fatal and survival patients with SSc–ILD

Clinical features	SS-ILD deceased (*N* = 11, 21.2%)		SS-ILD survived (*N* = 41, 78.8%)		*p*-value
	*n*/total (%)	Median(range)	*n*/total (%)	Median(range)	
Age (years)		59 (32–89)		61 (31–89)	0.814
Female (%)	7/11 (63.6)		35/41 (85.4)		0.190
Duration (year)		3 (0–24)		6 (0–32)	0.831
SSc onset age (year)		48 (24–77)		42 (12–74)	0.158
Smoking (%)	2/10 (20)		2/38 (5.2)		0.187
Overlapping with other autoimmune disease (%)	5/11 (45.5)		21/41 (51.2)		0.734
Digital ulcers (%)	4/11 (36.3)		17/41 (41.5)		0.760
PH (%)	9/11 (81.8)		11/34 (32.4)		0.001^*^
Cardiovascular disease (%)	5/11 (45.5)		6/37 (16.2)		0.043^*^
Raynaud’s phenomenon (%)	8/10 (80)		36/41 (87.8)		0.612
Dyspnea (%)	11/11 (100)		23/41 (56.1)		0.007^*^
ESR (mm/h)		46 (16–135)		27 (3–129)	0.025^*^
CRP (mg/dL)		3.5 (0.06–14.8)		0.40 (0.01–12.73)	0.017^*^
Hemoglobin (mg/dL)		12.8 (7.2–15.6)		12.5 (7.5–17)	0.823
Albumin (mg/dL)		3.1 (1.9–4.0)		3.9 (2.2–4.7)	0.005^*^
Creatinine (mg/dL)		1.05 (0.67–6.22)		0.71 (0.48–1.63)	0.003^*^
TLC of predicted (%)		63 (45–91)		81 (49–115)	0.011^*^
FVC of predicted (%)		53 (37–82)		70 (32–108)	0.088
FEV1 of predicted (%)		64 (34–97)		69 (27–110)	0.497
DLCO of predicted (%)		25 (19–46)		44 (17–79)	< 0.001^*^
Steroid (%)	9 (81.8)		25 (61.0)		0.197
Sildenafil citrate (%)	2 (18.2)		10 (24.4)		0.664
ANA (%)	8/9 (88.9)		35/40 (87.5)		0.909
Anti-Scl70 Ab (%)	3/10 (30)		19/36 (52.8)		0.202
Anti-RNP Ab (%)	2/9 (22.2)		7/23 (30.4)		0.642

*SSc* systemic sclerosis, *ILD* interstitial lung disease, *PH* pulmonary hypertension, *ESR* erythrocyte sedimentation rate, *CRP* C-reactive protein, *TLC* total lung capacity, *FVC* forced vital capacity, *FEV1* forced expiratory volume in one second, *DLCO* diffusing capacity of lung for carbon monoxide, *ANA* antinuclear antibody, *ab* antibodies. *p-value < 0.05

**Table 3:** Logistic regression analysis of clinical features in patients with SSc with ILD

Univariate	OR	95% CI	*p-*value
PH	18.81	2.11–167.70	0.009*
CVD	4.30	0.98–18.80	0.052
ESR	1.22	1.00–1.04	0.029*
CRP	1.23	1.02–1.48	0.026*
Hypoalbuminemia	0.23	0.07–0.71	0.01*
Creatinine	33.60	2.00–546.10	0.014*
TLC	0.93	0.88–0.99	0.021*
DLCO	0.86	0.78–0.95	0.003*

*PH* pulmonary hypertension, *ILD* interstitial lung disease, *CVD* cardiovascular disease, *ESR* erythrocyte sedimentation rate, *CRP* C-reactive protein, *TLC* total lung capacity, *DLCO* diffusing capacity of the lung for carbon monoxide. *p-value < 0.05

### Radiographic features in the lungs among SS–ILD patients

We compared the clinical data between SSc–ILD patients who showed usual interstitial pneumonia (UIP) and non-UIP patterns (Table 4). Non-UIP pattern includes nonspecific interstitial pneumonia (NSIP) and bronchiolitis obliterans with organizing pneumonia (BOOP). In 52 SSc–ILD patients, 33 were with UIP (63.5%) and 19 (36.5%) were with non-UIP. One patient had BOOP and 18 patients exhibited NSIP in the non-UIP group. The overlapping syndrome with other autoimmune diseases was significantly more frequent in the non-UIP group (73.7% vs. 33.4%, *p* = 0.011). Raynaud’s phenomenon (UIP vs. non-UIP: 84.9% vs. 84.2%), esophageal dysmotility (100% vs. 85.7%), GERD (89.3% vs. 86.7%), and digital ulcers (45.5% vs. 31.6%) were almost equally found in both groups. A tendency of longer disease duration (7 years vs. 4 years, *p* = 0.085) with deteriorated DLCO (35% vs. 46%, *p* = 0.096) in the UIP group was also observed but not reaching the statistically significant level. A comparison of the serological characteristics revealed that ANA titer (> 1:80, UIP vs. non-UIP: 83.9% vs. 94.4%, *p* = 0.271) and SSc-specific autoantibody Scl-70 (UIP vs. non-UIP: 48.3% vs. 47.1%, *p* = 0.936) were equally prevalent in both groups. The anti-U1-RNP autoantibodies were significantly more frequent in the non-UIP group (53.8% vs. 10.5%, *p* = 0.011).

**Table 4:** Clinical and laboratory features of UIP and non-UIP groups in 52 patients with SSc with ILD

Clinical features	UIP (*n* = 33, 63.5%)	Non-UIP (*n* = 19, 36.5%))	*p*-value
Age (years)	62 (33–89)	60 (31–89)	0.216
Female (%)	25/33 (75.8%)	17/19 (89.5%)	0.202
Duration (year)	7 (0–32)	4 (0–23)	0.085
SSc onset age (year)	43 (12–77)	43.5 (22–71)	0.859
BMI	19.6 (12.9–39.3)	20.5 (13.5–26.7)	0.645
Mortality (%)	7 (21.2%)	4 (21.1%)	0.638
Smoking (%)	2 (6.3%) (*n* = 32)	2 (12.5%) (*n* = 16)	0.407
Overlapping with other autoimmune disease (%)	12 (36.4%)	14 (73.7%)	0.011^*^
Digital ulcers (%)	15 (45.5%)	6 (31.6%)	0.326
PH (%)	15 (51.7%) (*n* = 29)	5 (33. 3%) (*n* = 15)	0.301
Cardiovascular disease (%)	9 (30%) (*n* = 30)	2 (11.1%) (*n* = 18)	0.123
Raynaud’s phenomenon (%)	28 (84.9%)	16 (84.2%)	0.525
Abnormal esophageal transit time (%)	13 (100%) (*n* = 13)	6 (85.7%) (*n* = 7)	0.350
GERD (%)	25 (89.3%) (*n* = 28)	13 (86.7%) (*n* = 15)	0.493
Dyspnea (%)	22 (66.7%)	12 (63.1%)	0.798
**Laboratory data**			
ESR (mm/h)	30.5 (3–135)	27 (7–129)	0.754
CRP (mg/dL)	0.72 (0.01–14.8)	0.62 (0.04–7.45)	0.037^*^
Hemoglobin (mg/dL)	12.8 (7.2–17)	12.3 (7.6–15)	0.562
Albumin (mg/dL)	3.7 (1.9–4.7)	3.9 (2.2–4.5)	0.571
Creatinine (mg/dL)	0.72 (0.48–6.22)	0.77 (0.54–2.21)	0.967
**Pulmonary functional test**			
TLC of predicted (%)	71 (45–115)	81 (55–115)	0.383
FVC of predicted (%)	60 (32–104)	72 (35–108)	0.684
FEV1 of predicted (%)	63 (27–99)	75 (39–110)	0.344
DLCO of predicted (%)	35 (17–65)	46 (20–79)	0.096
**Medication use**			
Steroid (%)	21 (63.6%)	13 (68.4%)	0.727
Revatio (%)	8 (24.2%)	4 (21.1%)	0.538
**Autoantibody**			
ANA (%)	26 (83.9%) (*n* = 31)	17 (94.4%) (*n* = 18)	0.271
Anti-ds DNA Ab (%)	5 (17.9%) (*n* = 28)	6 (35.3%) (*n* = 17)	0.168
Anti-Scl70 Ab (%)	14 (48.3%) (*n* = 29)	8 (47.1%) (*n* = 17)	0.936
Anti-RNP Ab (%)	2 (10.5%) (*n* = 19)	7 (53.8%) (*n* = 13)	0.011^*^
Anti-SSA/SSB Ab (%)	13 (52%) (*n* = 25)	5 (33.3%) (*n* = 15)	0.251

*SSc* systemic sclerosis, *BMI* body mass index, *PH* pulmonary hypertension, *GERD* gastroesophageal reflux disease, *ESR* erythrocyte sedimentation rate, *CRP* C-reactive protein, *TLC* total lung capacity, *FVC* forced vital capacity, *FEV1* forced expiratory volume in one second, *DLCO* diffusing capacity of lung for carbon monoxide, *ANA* antinuclear antibody, *Revatio* sildenafil citrate. ^*^p-value < 0.05

## Discussion

Our study on this single medical center cohort of SSc in Taiwan shows that the prevalence of ILD in SSc patients might range from 10.5% to 47.3%. Since Taipei VGH is the largest medical center in Taiwan with a usual active bed number of 3,136 and an annual outpatient number of 655,670 (in 2024), the statistical features obtained in the present investigation would reflect the *status quo* of SSc–ILD in this country. As expected, pulmonary function parameters were significantly different between the non-ILD and ILD groups. Consistently with those have been reported, FEV1, FVC, and TLC could well distinguish each group, and DLCO was significantly reduced in the SSc patients with ILD. Interestingly, we found a reverse correlation between TLC and inflammatory markers ESR and CRP. This reverse correlation is also observed in DLCO, FVC, and FEV1 and is consistent with published data [14]. It remains controversial whether the levels of acute phase reactants, such as CRP, correlate with the clinical outcome of scleroderma. Our data (as shown in Fig. 2) strongly suggest that the inclusion of ESR and CRP enhances the power of PFT in predicting ILD in patients with scleroderma and will aid clinicians in determining the need for chest computerized tomography (CT).

As analyzed by CT scan and other experimental modalities, there were almost twice the number of patients with UIP (63.5%) compared to those with non-UIP in SSc–ILD and high frequency of cancers in SSc patients with (23.1%) or without (27.6%) ILD as shown in Tables 4 and 1. Therefore, it is crucial to keep a high index of suspicion for early detection of ILD as well as malignancies in oriental SSc patients and to give them appropriate treatments as soon as possible for precluding them from developing overwhelming infections, especially because there are quite poor environmental factors in the vast Asia–Pacific region compared to the western countries, which may render the SSc patients prone to much more lethal opportunistic infections. When compared to the general population, some studies have observed the increased mortality rate in SSc patients [15–17]. In Taiwanese population, the SSc cases are also extraordinarily fatal with a standardized mortality ratio (SMR) being 3.24 [18]. In a French cohort study, the overall SMR in SSc patients is 5.73 [19]. Furthermore, SSc with pulmonary involvement is the leading cause of death [8, 16]. The overall mortality in the present SSc–ILD cohort was up to 30.9% (34/110). Interestingly, 23 out of 58 patients (39.7%) who had undergone pulmonary evaluations but were not found to have coexisting ILD conversely had a higher mortality rate. On the other hand, 11 out of 52 patients (21.2%) with SSc–ILD died. Apparently, the patients without ILD had a higher cancer rate, with more than one-third of them suffering from malignancies (exact data not shown). It is possible that the underlying neoplasms with immunocompromised status associated with some kind of lung diseases (including ILD) contributed to their death. Another nationwide population investigation in this country has also demonstrated a higher SMR contributed from neoplasms (SMR = 1.5) in SSc patients [18]. Among deceased SSc patients with ILD in the present investigation, 8/11 (72.7%) died mainly from infectious complications. In contrast, a meta-analysis has demonstrated the percentage of infection-related deaths is around 42% in SSc patients [20]. Another investigation suggested that infections in lower respiratory tracts constitute a risk factor for the deterioration of pulmonary function, especially in SSc patients with lung involvement [21]. Therefore, infections should be a more imperative concern, whatsoever the patients with ILD receive or not receive immunosuppressive medications [22]. In other words, our results imply a mandatory strict scrutiny for the occurrences of infections and neoplasia in patients with SSc.

Regarding clinical symptoms, dyspnea, fatigue, and non-productive cough are the most common manifestations of SSc–ILD [23]. In an analysis of SSc–ILD, patient-centered measures of the perceived dyspnea could be a way for accurately assessing and monitoring progression of the disease [24]. A systematic review has suggested that dyspnea is an independent predictor of mortality, especially in the heart and lung complications [25]. Khadawardi et al*.* reported that the assessment of dyspnea in patients with chronic ILD can reflect disease severity and may help predict the progression of disease as well as mortality [26]. In the present SSc–ILD cohort, all fatal SSc–ILD patients had an initial presentation of dyspnea. These results are consistent with those revealed previously. It is also worth noting that our data were retrieved until 2020, just before the COVID-19 pandemic. Because the COVID incidence in Taiwan was slightly later than the worldwide pandemic, the present data do not include pandemic materials and are purely autoimmune-based, which were not mixed up with SARS-CoV-2-induced ILD or various autoimmune diseases that were claimed to be induced by SARS-CoV-2 infection [27]. Thus, it is conceivable that even without influenza or any viral pandemic that may leave inflammation sequelae in the lungs, we should be very cautious about the clinical course of pulmonary function as well as constitutional symptoms when encountering SSc–ILD patients with dyspnea at the earlier stage of disease to prevent poor outcomes.

It is well known that PH is a major impact on the survival of patients with SSc [28]. In the present study, we have also demonstrated the clinical pertinence of PH in relation to mortality in SSc–ILD although we could only recognize PH by echocardiography rather than cardiac catheterization because of the reluctance of patients or their families or the critical condition of most of our patients unfeasible to undergo the riskier cardiac catheterization procedure. Nine out of 11 (81.8%) fatal SSc patients with ILD had PH, which was terribly prevalent. In contrast, only 32.4% of the surviving SSc–ILD patients had PH. So, the entity is certainly one of the important risk factors for death as calculated by univariate analysis. On the other hand, reductions in FVC, TLC, and DLCO have been identified in previous investigations as predictors for the poor outcomes of SSc–ILD [29–31]. Our present data have also indicated that decreased TLC and DLCO are closely associated with mortality in the SSc–ILD cohort. These results are consistent with previous reports and highlight the prognostic values of pulmonary function measurements in SSc–ILD. A part of these patients with SSc–ILD-associated PH had already received endothelin receptor antagonist. Therefore, ILD seemed to account for a higher percentage of deaths. Based on univariate analysis, deterioration of renal function is also a risk factor for mortality in our SSc–ILD patients. It is thus conceivable that PH, pulmonary, and renal dysfunctions, as well as the corresponding deteriorations, contribute well to the poor outcome of SSc–ILD, which is compatible with those published previously [4, 32]. Whether these predisposing factors are influenced by each other and synergistically result in the accelerated deterioration of SSc deserves further study with larger cohorts.

Anti-Scl-70 antibodies occur in SSc patients with a frequency of approximately 28% (9.4 to 42%) [33], and the presence of anti-Scl-70 antibodies is a major risk factor for the development of ILD in SSc patients [34]. It is highly specific for SSc and is associated with a higher risk for pulmonary fibrosis [35, 36]. Anti-SSA/SSB antibodies are detectable in up to 15–20% of patients with SSc and may also confer hazards for SSc–ILD [37]. In the present study, we could only demonstrate a non-significant trend towards more frequent presence of anti-Scl-70 and anti-SSA/SSB autoantibodies in SSc patients with ILD, and there was no survival difference among them. Since it is difficult to enroll more patients with SSc–ILD because of its rarity in Taiwan, we are unable to collect enough number of patients to validate the significance of SSA/SSB antibodies in predicting outcomes of SSc–ILD, and a more comprehensive study is necessary on a larger database such as those deposited in nationwide health administration.

Regarding the CT images, NSIP and UIP are two main types of ILD in SSc. Previous studies have disclosed that the NSIP pattern is more frequent, being present in about 78% of SSc patients [38, 39]. On the other hand, the UIP pattern is associated with a lower survival rate in SSc–ILD [40, 41]. In the present study, we observed a higher frequency of UIP (63.5%) in SSc–ILD, but the mortality in UIP and non-UIP was not significantly different. Longer disease duration, less overlapping syndrome, and higher CRP were observed in the UIP group. Thus, we propose that the major risk factors associated with mortality might be the presence of PH and poor pulmonary function parameters rather than the individual ILD patterns, which is compatible with Bouros’ study [42].

Our study also showed a higher frequency of anti-U1-RNP autoantibodies in the non-UIP group. Anti-U1-RNP is a pathogenic antibody for MCTD and SLE [43]. In our data, more SLE and MCTD patients were present in the non-UIP group, who constituted the most frequent co-existing diseases in SSc–ILD (Supplementary Table S2). Since the anti-U1-RNP antibody has been found significantly associated with vascular endothelial dysfunction and Raynaud’s phenomenon, endothelial dysfunction may play a significant pathogenic role in the development of non-UIP rather than UIP. However, further larger observational research or in vitro endothelial cell culture interfered with U1-RNP antibody may be required to understand the significance of anti-U1-RNP autoantibodies in the pathogenesis of SSc–ILD subgroup patterns such as NSIP or BOOP.

In contrast to those reported in the similar investigations on SSc–ILD in western countries, the frequency of ILD in the present Taiwanese SSc patients can be as high as 47.3%. This high figure ranged between those found in Canada and UK studies (32.6% and 60%) [3, 4]. However, as mentioned above, our estimation may be falsely high because this calculation excluded those who did not undergo chest radiograph, pulmonary function test, or CT scan (largely patients lost to follow-up). Yet, if the strictly measured prevalence of 10.5% is true, this figure still indicates a high incidence of ILD in our SSc patients. Furthermore, we have demonstrated that the major risks of mortality were the presence of PH and poor pulmonary function parameters rather than the individual ILD patterns. Besides, early-onset dyspnea and poor renal function are also independent risk factors for mortality in the SSc–ILD.

Inevitably, the present investigation holds some limitations. For the first, it is limited by the analysis of patients from a single tertiary care center, although it is the largest single retrievable source of SSc–ILD patients in this country, as has been mentioned above. For the second, the study was restrained by a relatively small sample size with lower statistical power to identify subtle predictors of outcomes because of an intrinsic defect of scarceness of SSc in Taiwan. For the third, this is a retrospective cross-sectional study lacking long-term follow-up periods. For the fourth, there had been still no official authorization (indication) for prescribing anti-fibrotic agents (including nintedanib, pirfenidone) to treat SSc–ILD as early as possible at the beginning of patient enrollment in this study (April 2000). Therefore, we have no well-logged medical records regarding anti-fibrotic agents for SSc–ILD patients to carry out further investigations. Finally, its retrospective property may have resulted in missing or inadequately collecting data from the EMRs. It was impossible to assess certain known predictive factors for ILD progression, such as SSc disease activity. Despite these limitations and difficulties, we still reached a conclusion that early recognition of SSc–ILD is challenging because of the lack of specific symptoms. Based on the similar geographic, environmental, and ethnic characteristics in Asian countries, we might suggest that our results be extrapolated to the SSc patients in the vast area of poor sanitary conditions in the Asia–Pacific territory.

In conclusion, this retrospective study, encompassing two decades, has shown ILD as a crucial complication in Taiwanese SSc patients. Infections and malignant neoplasms contribute remarkably to the mortality in these patients. Dyspnea, PH, and poor renal function rather than different ILD patterns may synergistically or individually pose a risk for death in SSc–ILD patients. However, more future studies are needed to clarify the underlying pathogenic mechanisms that drive the liable development of ILD in oriental SSc such as our retrospective cohort, thus facilitating a more effective treatment strategy in Asian patients with SSc. Furthermore, whether the COVID-19 pandemic permanently changed the pattern of ILD manifestations associated with autoimmune diseases including SSc also deserves future investigations.

## Supplementary Information

Below is the link to the electronic supplementary material.

ESM1 (DOCX 103 KB)

## Data Availability

All data relevant to the present investigation are included in the article. Data are available upon reasonable request. Encrypted primary data may be available from the corresponding author upon reasonable request.

## Abbreviations

ACR: American College of Rheumatology
ANA: Antinuclear antibodies
AUC: Area under the curve
BOOP: Bronchiolitis obliterans and organizing pneumonia
CHF: Congestive heart failure
CI: Confidential interval
COVID-19: Coronavirus disease 2019
CRP: C-reactive protein
CT: Computerized tomography
CVD: Cardiovascular disease
DLCO: Diffusion capacity of carbon monoxide
dsDNA: Double-stranded deoxyribonucleic acid
EMR: Electron medical records
ESR: Erythrocyte sedimentation rate
EULAR: European League Against Rheumatism
EUSTAR: European League Against Rheumatism Scleroderma Trials and Research
FEV1: Forced expiratory volume in one second
FVC: Forced vital capacity
GERD: Gastro-esophageal reflux disease
GID: Gastrointestinal disease
Hb: Hemoglobin
HRCT: High-resolution computed tomography
ICD-9-CM or ICD-10-CM: International Classification of Diseases, Ninth Revision or Tenth Revision, Clinical Modification
ILD: Interstitial lung disease
MCTD: Mixed connective tissue disease
NSIP: Nonspecific interstitial pneumonia
OR: Odds ratio
PFT: Pulmonary function test
PH: Pulmonary hypertension
ROC: Receiver operating curve
RP: Raynaud’s phenomenon
RVSP: Right ventricular systolic pressure
Scl70: Scleroderma autoantigen of 70 kilodaltons or topoisomerase I
SLE: Systemic lupus erythematosus
SMR: Standardized mortality ratio
SPSS: Statistical Package for the Social Sciences®
SS-A or SS-B: Sjögren’s syndrome autoantigen A or B
SSc: Systemic sclerosis
TLC: Total lung capacity
TVGH: Taipei Veterans General Hospital
U1-RNP: Nuclear ribonucleoprotein 1
UIP: Usual interstitial pneumonia
