# Supplementary material for: Interstitial lung disease in systemic sclerosis—a retrospective cross-sectional study in Taiwan
Source: Clin Rheumatol. 2026 Jan 7;45(2):1011–22. doi: 10.1007/s10067-025-07913-y (PMC12858559; doi:10.1007/s10067-025-07913-y)
Supplement: Supplementary file 1 — (DOCX 103 KB) [file 10067_2025_7913_MOESM1_ESM.docx]

**Supplemental tables**

**Table S1** Cause of death in 34 fatal patients with systemic sclerosis

| Cause of death | Total SSc (n=34) | SSc-ILD  (n=11) | SSc without ILD (n=23) |
| --- | --- | --- | --- |
| Infection | 15 | 8 | 7 |
| Malignancy | 10 | 2 | 8 |
| CV events | 7 | 1 | 6 |
| Cardiogenic shock | 4 | 1 | 3 |
| Heart failure | 1 | 0 | 1 |
| Acute coronary syndrome | 1 | 0 | 1 |
| Pulmonary arterial hypertension | 1 | 0 | 1 |
| Massive bleeding in GI tract | 2 | 0 | 2 |

SSc: systemic sclerosis, ILD: interstitial lung disease, CV: cardiovascular, GI: gastrointestinal.

*Respiratory failure is not included as a cause of death because in all of the patients, individual cause of death was more or less relevant or synergistic to the underlying cause.

**Table S2** Systemic sclerosis patients overlapped with other autoimmune diseases


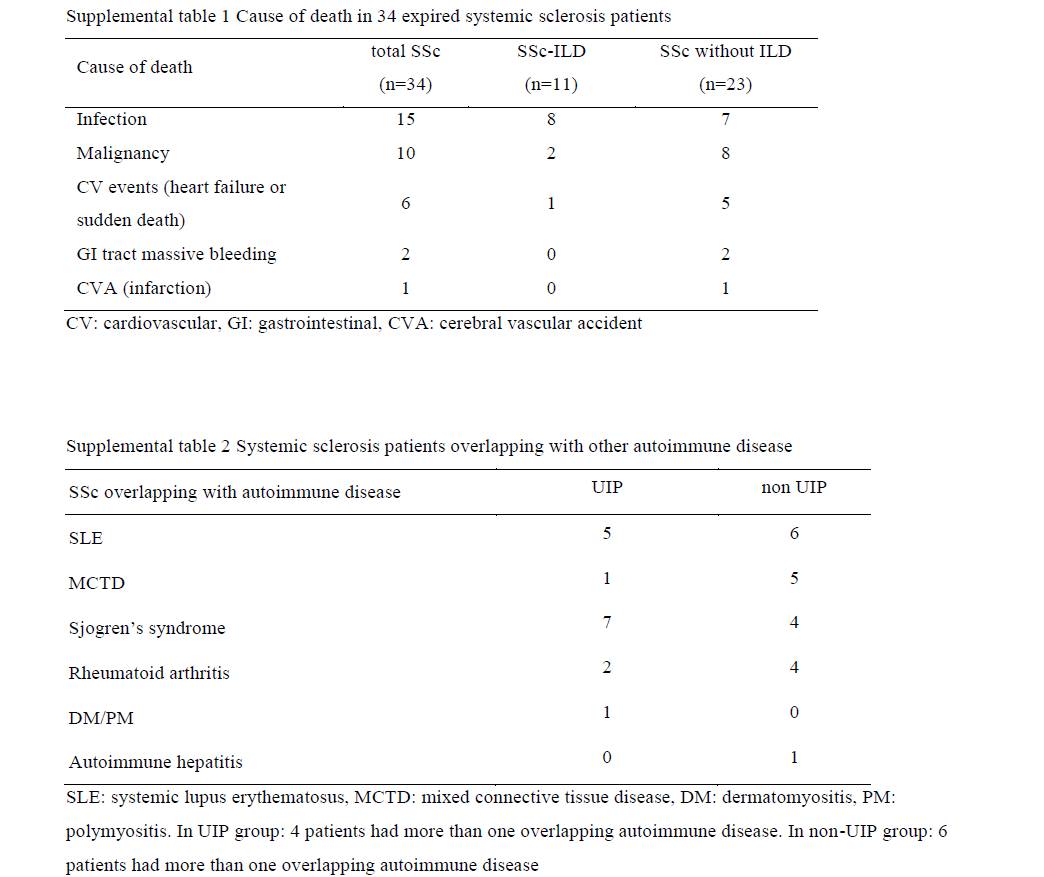


UIP: usual interstitial pneumonia, SLE: systemic lupus erythematosus, MCTD: mixed connective tissue disease, DM: dermatomyositis, PM: polymyositis.
